# More distinct food intake patterns among women than men in northern Sweden: a population-based survey

**DOI:** 10.1186/1475-2891-8-12

**Published:** 2009-02-19

**Authors:** Anna Winkvist, Agneta Hörnell, Göran Hallmans, Bernt Lindahl, Lars Weinehall, Ingegerd Johansson

**Affiliations:** 1 Department of Clinical Nutrition, Sahlgrenska Academy, University of Gothenburg, Göteborg, Sweden; 2 Department of Food and Nutrition, Umeå University, Umeå, Sweden; 3 Nutrition Research, Department of Public Health and Clinical Medicine, Umeå University, Umeå, Sweden; 4 Behavioral Medicine, Department of Public Health and Clinical Medicine, Umeå University, Umeå, Sweden; 5 Department of Community Medicine, Västerbotten County Council, Umeå, Sweden; 6 Epidemiology and Public Health Sciences, Department of Public Health and Clinical Medicine, Umeå University, Umeå, Sweden; 7 Cariology, Department of Odontology, Umeå University, Umeå, Sweden

## Abstract

**Background:**

The need to promote a healthy diet to curb the current obesity epidemic has today been recognized by most countries. A prerequisite for planning and evaluating interventions on dietary intake is the existence of valid information on long-term average dietary intake in a population. Few large, population-based studies of dietary intake have been carried out in Sweden. The largest to date is the Västerbotten Intervention Program (VIP), which was initiated in 1985, with data collection still ongoing. This paper reports on the first comprehensive analyses of the dietary data and presents dietary intake patterns among over 60,000 women and men in northern Sweden during 1992–2005.

**Methods:**

Between 1992 and 2005, 71,367 inhabitants in Västerbotten county aged 30, 40, 50, and 60 years visited their local health care center as part of the VIP. Participants of VIP filled in an 84- or 64-item food frequency questionnaire (FFQ) and provided sociodemographic information. Complete and realistic information on consumption frequency was provided by 62,531 individuals. Food intake patterns were analyzed using K-means cluster analyses.

**Results:**

The mean daily energy intake was 6,83 (± 1,77) MJ among women and 8,71 (± 2,26) MJ among men. More than half of both women and men were classified as Low Energy Reporters (defined as individuals reporting a food intake level below the lower 95% confidence interval limit of the physical activity level). Larger variation in frequency of daily intake was seen among women than among men for most food groups. Among women, four dietary clusters were identified, labeled "Fruit and vegetables", "High fat", "Coffee and sandwich", and "Tea and ice cream". Among men, three dietary clusters were identified, labeled "Fruit and vegetables", "High fat", and "Tea, soda and cookies".

**Conclusion:**

More distinct food intake patterns were seen among women than men in this study in northern Sweden. Due to large proportions of Low Energy Reporters, our results on dietary intake may not be suitable for comparisons with recommended intake levels. However, the results on food intake patterns should still be valid and useful as a basis for targeting interventions to groups most in need.

## Background

Chronic diseases are on the rise worldwide. At the risk factor level, these can be regarded as communicable diseases in so far as major risk factors such as diet and physical activity patterns are spreading from culture to culture, thus changing the disease pattern globally [1]. Convincing evidence exists for associations between high intake of energy-dense foods, as well as low intake of fruits and vegetables, and obesity as well as cardiovascular diseases [1]. Convincing evidence also exists for associations between high intake of sugars, and dental caries; low intake of vitamin D, and osteoporosis; high intake of red meat and processed meat, and colorectal cancer; and high intake of alcohol, and cancer of the oral cavity, pharynx, larynx, esophagus, and breast [1,2].

The need to promote a healthy diet to curb the obesity epidemic has today been recognized by most countries. In 2004, the World Health Organization presented its *Global Strategy on Diet, Physical Activity and Health*, which urges all United Nations member states to promote lifestyles that include a healthy diet and foster energy balance [3]. Similar plans of action on diet and physical activity have recently been adopted by European as well as Nordic countries [4,5].

A prerequisite for planning and evaluating interventions on dietary intake is the existence of valid information on long-term average dietary intake as well as patterns of intake in a population. Few large, population-based studies of dietary intake have been carried out in Sweden. The largest to date is the Västerbotten Intervention Program (VIP), which was initiated in 1985, with data collection still ongoing. This paper reports on the first comprehensive analyses of the dietary data and presents dietary intake patterns among over 60,000 women and men in northern Sweden during 1992–2005.

## Methods

### Study population

Since 1985, inhabitants in Västerbotten county in northern Sweden (population of 255,000) have been invited by a mailed invitation letter to their local health center for a medical examination when turning 40, 50, and 60 years of age. Initially, also 30-year olds were invited. Participants fill in an extensive diet and lifestyle questionnaire. During 1992–1993, 57 percent of eligible inhabitants participated and there were no systematic differences between participants and non-participants [6]. Participation rates have since been 58–66 percent.

### Food intake measurements

The questionnaire includes a semi-quantitative food frequency questionnaire (FFQ). Portion sizes are indicated by color photographs of four plates of increasing portion sizes for staple food, meat, and vegetables. Frequency of dietary intake is reported on a 9-level scale, from "none" to "four or more times a day". Initially, the FFQ included 84 food items, but from 1996 a 64 food item version was introduced. The reduction to 64 food items was achieved by deleting entire foods or, in a few cases, by merging similar food items. Among the participants included here, 59.2% of the 40, 50 and 60 yrs old participants had filled in the 64-food item version and 40.8% the 84-food item version. Among the 30 yrs old participants, 16.3% had filled in the 64-food item version and 83.7% the 84-food item version, reflecting participation only in the early phase of VIP. Women had a reported energy intake of 7.45 (± 1.84) MJ for the 84-food item version and 6.38 (± 1.58) MJ for the 64-food item version (P < 0.000). Men had a reported energy intake of 9.01 (± 2.23) MJ for the 84-food item version and 8.51 (± 2.25) MJ for the 64-food item version (P < 0.000). These two sources of dietary data have been combined into one file for the purpose of the food patterns analyses, after harmonizing both data sources. In this process, food items in the 84-food item version were merged to correspond with similar food item groups in the 64-food item version.

Daily intake has been calculated by multiplying frequency of intake by a portion size value using the national food composition database [7]. Portion sizes used were those indicated on the photographs, natural sizes such as an orange, or average portion sizes for the sex and age as determined in a national survey [8,9]. Energy and nutrient contents were calculated using the software MATs (Rudans Lättdata, Västerås, Sweden).

A validation of the 84-item FFQ was carried out among 246 participants, using ten repeated 24 hour dietary recalls and plasma β-carotene [9]. These participants repeated the FFQ one year later to assess reliability. Good agreement was found for energy and nutrients between the two occasions (median Pearson correlation 0.68). A moderately higher dietary intake was found for the FFQ compared with the 24 hour dietary recall with respect to dairy products, bread/cereals, vegetables, fruits, and potato/rice/pasta. By contrast, a moderately lower dietary intake was found for meat, fish, sweet snacks, and alcohol. The average Spearman's correlation coefficient between the two methods was 0.15–0.69 for various foods and 0.31–0.61 for various nutrients, indicating poorer ability of the FFQ to accurately estimate levels of nutrient intake. However, more than 68 percent of the participants placed within the lowest and highest FFQ quartiles were classified also into the corresponding quartiles of the 24 hour dietary recalls for both foods and nutrients. Extreme misclassification between the lowest and highest quartiles with respect to any food or nutrient was noted for fewer than five individuals. Also, the measurements in the 24 hour dietary recalls increased monotonically over the quartiles of the FFQ measurement for all foods and most nutrients. Hence, the FFQ should be valid for the ranking of individuals with regard to food and nutrient intake.

In total, 83,013 health checkups (43,128 in women and 39,885 in men) were performed 1992 – 2005. Information provided before 1992 was not optically readable and was therefore not available. Among the 83,013 visits, 71,367 were first-time visits and only these were included here. Among these 71,367 individuals, information on portion sizes was missing for 1,747 individuals and information on consumption frequency was missing on more than 10% of the food items for 1,293 individuals. Food intake level (FIL) was calculated as reported total caloric intake, divided by estimated basal metabolic rate (BMR) [10]. Altogether, 5,425 individuals with an FIL below the 5th percentile or above the 97.5th percentile (calculated separately by sex and FFQ version) were deemed unrealistic and excluded. Another 371 individuals lacked FIL information. The final sample included only individuals with acceptable data on portion sizes, intake frequencies, and FIL (n = 62,531; 32,600 women and 29,931 men).

To assess levels of low energy reporting, the FIL was compared with physiologically plausible physical activity levels (PALs), calculated as the ratio of total energy expenditure to BMR [11]. Confidence interval limits for agreement between FIL and PAL were calculated as follows:

FIL > PAL × exp [SD × (S/100)/√*n*],

where *SD *is the number of SDs corresponding to the confidence interval (CI) chosen, *S *is the overall coefficient of variation for PAL, taking into account the variability in energy intake and BMR, and *n *is the number of subjects included. *S *was calculated as follows:

S = √(CW^2^_IW_/k + CV^2^_B _+ CV^2^_P_)

where *CW*^*2*^_*IW *_is the within-individual variation in energy intake, *k *is the number of measurement days, *CV*^*2*^_*B *_is the precision of estimated vs. measured BMR, and *CV*^*2*^_*P *_is the between-subject variation in PAL. In our analyses, *CW*_*IW *_equaled 28.6% and was derived from the ten 24 hour dietary recalls in our validation study [9]; *CV*_*B *_was set to 8% [10], and *CV*_*P *_was assumed to be 12.5% [12]. Furthermore, *n *equaled one individual, and *k *for food frequency = ∞, meaning that *CW*^*2*^_*IW*_/*k *= 0. The SD was set to -1.96 to obtain the lower limit of a 95% CI. The PAL was estimated from reported physical activity at work and at leisure [13]. Accordingly, the lower 95% CI limit of the FIL corresponded to PAL × 0.748 and this was used to define Low Energy Reporters on an individual level. Similarly, the upper 95% CI limit corresponded to PAL × 1.336 and this was used to define High Energy Reporters.

### Data analyses

Food intake patterns were evaluated using cluster analysis of observations, which separates participants into mutually exclusive groups and maximizes differences in intake of a number of food groups. The QUICK CLUSTER procedure in SPSS, version 14.0 (Chicago, IL, USA), was used, based on the K-means method. This procedure organizes individuals into optimal clusters, starting with the initial individual in the data file and thereafter adding individuals one by one. To minimize any effect of the ordering of individuals, five random variables were created and used to sort the data file in ascending as well as descending order. Cluster assignment was robust for both women and men across the ten runs obtained; 97 to 99% of the women and 92 to 100% of the men were assigned to the same cluster in all runs. Emerging patterns were evaluated for two to ten clusters for women and men, respectively. Differences among the clusters in intake of all included food groups were scrutinized and the F-statistic for the analysis of variance tables was inspected. Food items from the FFQ were grouped into meaningful food groups according to nutrient content and/or culturally relevant culinary preferences. Finally, 36 food groups were included (Table 1). Dietary data based on frequency of intake were used in the analyses. To improve robustness of the analyses the frequency of intake was energy adjusted by using frequency/1000 kcalories. The cluster analyses also were repeated after removing all Low Energy Reporters. Groups with similar unique dietary intake characteristics to those reported for the whole sample were found among both women and men; only exception was lower coffee intake among women in the group labeled "Coffee and sandwich" and lower tea intake among men in the group labeled "Tea, soda and cookies". Finally, the cluster analyses were repeated on the 64-item and the 84-item FFQs separately, and the patterns were stable across the two versions.

**Table 1:** Food items included in the 36 food groups used in cluster analyses

**Food group**	**Food items**
High-fat spreads	Butter and high-fat margarine (80% fat)
Low-fat spreads	Low-fat margarine (40% fat)
Oil	Vegetable oils in cooking and as salad dressing
Fat in cooking	Butter and margarine used in cooking
Fruit	All fruits and berries (fresh and frozen)
Vegetables	All vegetables (fresh and frozen)
Milk, 0.5%	Milk, fermented milk 0.5% fat
Milk, 1.5%	Milk, fermented milk 1.5% fat
Milk, 3%	Milk, fermented milk 3.0% fat
Cream	Cream, sour cream, creme fraiche
High-fat cheese	Cheese, hard, 28% fat
Low-fat cheese	Cheese, hard, 17% fat
Cereals	Cold cereals
White bread	White bread, soft and hard
High fiber bread	High fiber bread, soft and hard
Boiled potato	Boiled and mashed potato
Fried potato	Fried potatoe and French fries
Rice	Rice
Pasta	Pasta, makaroni
Fish	High fat and lean fish, shellfish
Red meat	Minced meat, stew, steak
Traditional meat	Bacon, sausage, hamburger
Chicken	Chicken, hen
Cold cuts	Meat, sausage and liverwurst on sandwich
Classic	Baked beans, pancakes, dumplings
Sweets	Candies, chocolate
Sugar and jam	Sugar, marmalade, jam, honey
Ice cream	Ice cream
Cookies	Cookies, cakes
Snacks	Chips, popcorn, peanuts
Soda	Sodas
Coffee	Coffee (boiled and filtered)
Tea	Tea
Beer	All types of beer
Wine	Red and white wine
Spirits	All types of spirits

Differences in background characteristics between included and excluded individuals as well as between Low Energy Reporters and normal reporters were evaluated using Student's *t*-test and chi-square test. Differences in food consumption patterns between women and men were investigated using the Mann-Whitney U-test, and differences among clusters within each sex were investigated using the Kruskal-Wallis analysis of variance test. This project was approved by the regional ethical committee, Göteborg, Sweden.

## Results

### Study population

Important differences between the 62, 531 included and 8, 836 excluded individuals included age [47.6 (± 9.3) vs. 49.3 (± 10.2) yrs, *P *< 0.001]; body mass index [25.7 (± 4.0) vs. 26.6 (± 5.0), *P *< 0.001]; marital status (81.2% married/cohabitating vs. 73.0% married/cohabitating, *P *< 0.001), education (25.0% basic level education only vs. 38.4% basic level education only, *P *< 0.001) and smoking (17.4% current smokers vs. 21.4% current smokers, *P *< 0.001).

Information on socio-demographic characteristics and dietary intake of the included individuals is given in Table 2. Among women, 19.0% were current smokers and 5.1% were current snuff users. Among men, 15.7% were current smokers and 26.8% were current snuff users.

**Table 2:** Basic characteristics of participants in the Västerbotten Intervention Program during 1992–2005

**Characteristic**	**Women** (n = 32, 600)	**Men** (n = 29, 931)
Age (yrs)	47.5 ± 9.3	47.6 ± 9.3
Height (cm)	164.7 ± 6.2	178.2 ± 6.8
Weight (kg)	68.7 ± 12.1	83.2 ± 12.2
Body mass index (kg/m^2^)	25.3 ± 4.4	26.2 ± 3.6
Marital status (%)		
Married/cohabitating	81.8	80.5
Unmarried/other	18.2	19.5
Education (%)		
Basic level (9 yrs)	24.4	25.7
High school	46.4	52.8
University	29.2	21.5
Activity level at work (%)		
Sedentary	23.2	25.4
Light	41.7	40.5
Moderate	28.9	28.4
Heavy	6.2	5.6
Activity level at leisure (%)		
Sedentary	44.3	44.2
Light	24.3	26.2
Moderate	19.0	15.0
Active	8.6	10.0
Highly active	3.9	4.5
Reported energy intake (MJ/d)	6.83 ± 1.77	8.71 ± 2.26
Macronutrient intake (% of total energy intake)		
Protein	15.1 ± 0.02	14.5 ± 0.02
Carbohydrates	51.7 ± 0.06	48.1 ± 0.06
Fat	31.8 ± 0.06	35.0 ± 0.06
Alcohol	0.01 ± 0.01	0.02 ± 0.02
Low Energy Reporters (%)	59.1	60.4
High Energy Reporters (%)	0.3	0.1

The proportion of different macronutrients of total energy intake was similar for both sexes. The proportion of individuals defined as Low Energy Reporters was high for both sexes. For both sexes, low energy reporting was significantly more common with higher BMI, higher age, and lower education (*P *< 0.05). Also, low energy reporting was significantly more common with higher levels of physical activity both at work and at leisure (*P *< 0.05), which probably reflects that energy reporting is evaluated in relation to level of physical activity. Reported intake of macro- and micronutrients decreased with increasing age among both women and men [see Additional file 1].

### Dietary patterns

Among the women, four distinct food intake pattern clusters were identified and these were labeled "Fruit and vegetables", "High fat", "Coffee and sandwich", and "Tea and ice cream". Among men, three distinct clusters were identified and were labeled "Fruit and vegetables", "High fat", and "Tea, soda and cookies". In Tables 3 and 4, intake of the 36 contributing food groups is indicated for these clusters as variation above and below the mean frequency of daily intake for each of the 36 food groups. The last column gives the overall mean frequency of intake per day for all women (Table 3) and men (Table 4).

**Table 3:** Food intake patterns in women in northern Sweden, 1992–2005

	**Food intake clusters**^1^				
**Food group**	Fruit and vegetables	High fat	Coffee and sandwich	Tea and ice cream	Overall mean (SD) frequency/day
Number of subjects (%)	4,729 (15)	10,572 (32)	9,138 (28)	8,161 (25)	32,600 (100)

High-fat spreads	-	+++	--	-	0.66 (0.75)
Low-fat spreads	-	--	+++	-	0.63 (0.77)
Oil	+	-	-	+	0.28 (0.36)
Fat in cooking	-	+	+	-	0.47 (0.41)
Fruit	+++	-	-	-	1.14 (0.74)
Vegetables	+++	-	-	-	1.19 (0.80)
Milk, 0.5%	+	-	+	-	0.39 (0.55)
Milk, 1.5%	-	+	+	-	0.46 (0.54)
Milk, 3%	-	+	-	=	0.32 (0.36)
Cream	-	+	=	=	0.10 (0.11)
High-fat cheese	-	+	=	=	0.43 (0.43)
Low-fat cheese	+	-	+	-	0.24 (0.36)
Cold cereals	+	-	+	=	0.33 (0.31)
White bread	-	+	+	-	0.39 (0.38)
High fiber bread	+	-	+	-	1.29 (0.72)
Boiled potato	+	=	=	-	0.40 (0.24)
Fried potatoes	-	+	+	=	0.06 (0.05)
Rice	+	-	=	+	0.11 (0.11)
Pasta	+	-	=	+	0.14 (0.10)
Fish	+	-	=	=	0.17 (0.10)
Red meat	+	-	=	=	0.30 (0.13)
Traditional meat	-	=	=	=	0.15 (0.07)
Chicken	++	-	=	=	0.06 (0.04)
Cold cuts	=	-	+	-	0.33 (0.31)
Classic	=	=	=	=	0.11 (0.06)
Sweets	-	+	=	+	0.14 (0.16)
Sugar and jam	-	+	=	-	0.40 (0.52)
Ice cream	=	=	=	+	0.06 (0.06)
Cookies	-	+	+	-	0.36 (0.33)
Snacks	-	=	=	=	0.05 (0.05)
Soda	-	+	=	+	0.19 (0.26)
Beer	=	+	+	=	0.05 (0.07)
Wine	=	-	=	-	0.05 (0.06)
Spirits	=	=	=	=	0.02 (0.03)
Coffee	+	+	+++	---	1.66 (0.95)
Tea	-	-	-	++	0.43 (0.54)

SD, Standard deviation

^1 ^The sign – indicates variation below the mean daily frequency of intake for all women, while + indicates variation above the mean daily frequency of intake for all women. For example, we used + (or -) for 0.1–0.49 SD units (SDUs); ++ (or --) for 0.5–0.99 SDUs; and +++ (or ---) for 1.0–1.99 SDUs. The sign = means equal to mean frequency of intake. For example, for "High-fat spreads", the "Fruit and vegetables" group had a mean intake that was 0.1–0.49 SDUs below the mean intake for all women, i.e., 0.1 × 0.75 – 0.49 × 0.75 below 0.66 times/day, which equals between 0.29 and 0.58 times/day.

**Table 4:** Food intake patterns in men in northern Sweden, 1992–2005

	**Food intake clusters**^1^			
**Food group**	Fruit and vegetables	High fat	Tea, soda and cookies	Overall mean (SD) frequency/day
Number of subjects (%)	9,179 (31)	8,725 (29)	12,027 (40)	29,931 (100)

High-fat spreads	--	++	+	0.70 (0.64)
Low-fat spreads	+++	--	--	0.41 (0.59)
Oil	+	-	=	0.18 (0.23)
Fat in cooking	-	+	=	0.33 (0.29)
Fruit	+	-	=	0.57 (0.45)
Vegetables	+	-	-	0.61 (0.45)
Milk, 0.5%	+	-	-	0.25 (0.41)
Milk, 1.5%	-	+	-	0.40 (0.46)
Milk, 3%	-	+	+	0.26 (0.32)
Cream	=	=	=	0.07 (0.08)
High-fat cheese	=	-	=	0.32 (0.32)
Low-fat cheese	+	-	-	0.16 (0.25)
Cold cereals	+	-	+	0.23 (0.23)
White bread	=	-	=	0.39 (0.35)
High fiber bread	+++	=	-	0.99 (0.59)
Boiled potato	=	=	-	0.29 (0.20)
Fried potatoes	+	=	=	0.07 (0.06)
Rice	=	=	=	0.08 (0.09)
Pasta	=	=	=	0.11 (0.08)
Fish	=	=	=	0.13 (0.07)
Red meat	=	=	=	0.25 (0.11)
Traditional meat	=	=	=	0.14 (0.07)
Chicken	+	=	=	0.04 (0.03)
Cold cuts	+	=	-	0.28 (0.26)
Classic	=	=	=	0.09 (0.05)
Sweets	=	-	=	0.11 (0.12)
Sugar and jam	-	+	-	0.52 (0.57)
Ice cream	=	=	=	0.05 (0.05)
Cookies	-	-	=	0.29 (0.27)
Snacks	=	=	=	0.04 (0.05)
Soda	-	-	+	0.19 (0.23)
Beer	=	+	=	0.09 (0.11)
Wine	+	=	=	0.03 (0.05)
Spirits	=	=	=	0.03 (0.04)
Coffee	+	++	--	1.36 (0.76)
Tea	=	-	+	0.26 (0.37)

SD, Standard deviation

^1 ^The sign – indicates variation below the mean frequency of intake, while + indicates variation above the mean frequency of intake. For example, we used + (or -) for 0.1–0.49 SD units (SDUs); ++ (or --) for 0.5–0.99 SDUs; and +++ (or ---) for 1.0–1.99 SDUs. The sign = means equal to mean frequency of intake. For example, for "High-fat spreads", the "Fruit and vegetables" group had a mean intake of 0.5–0.99 SDUs below the mean intake, i.e., 0.5 × 0.64 – 0.99 × 0.64 below 0.70 times/day, which equals 0.07–0.38 times/day.

In general, women had significantly more frequent consumption of fruits and vegetables compared with men (*P *< 0.000). Among the women's clusters, the average daily frequency intake of fruit and vegetables ranged from 0.91 to 2.00 and from 0.91 to 2.43 times per day, respectively. Among the men's clusters, the average daily intake of fruit and vegetables ranged from 0.49 to 0.65 and from 0.53 to 0.72 times per day, respectively. Also, women significantly more often consumed staple foods, bread, cheese, fish, chicken, sweets, wine, coffee, and tea compared with men (*P *< 0.000). By contrast, men consumed sugar and jam, beer, and spirits significantly more often compared with women (*P *< 0.000). Overall, larger variation in intake was seen among women than among men. This was indicated by the number of clusters identified as well as the larger SD in mean frequency of daily intake for most food groups among women than among men (Tables 3 and 4).

Consumption patterns for food groups and nutrients differed significantly among the clusters for both sexes (*P *< 0.000). The women in the "Fruit and vegetables" group typically had more frequent consumption of fruit and vegetables, chicken, fish, and red meat, compared with the other groups. Of the four groups, this group had the highest intake of carotenoids and vitamin C and the lowest intake of energy, fat, iron and retinol (data not shown). Typical for the women in the "High fat" group was a more frequent consumption of high-fat spreads, high-fat milk and high-fat cheese and cream. These women had the highest intake of fat (data not shown). Women in the "Coffee and sandwich" group reported more frequent consumption of coffee, bread, low-fat spreads, low-fat cheese and cold cuts. Finally, the women in the "Tea and ice cream" group had more frequent consumption of tea and ice cream, resulting in the highest intake of energy, protein, carbohydrates, calcium, and iron among the groups (data not shown).

The men in the "Fruit and vegetables" group had a more frequent consumption of fruits and vegetables, low-fat spreads, high fiber bread, low-fat milk and low-fat cheese, chicken, and wine. These men showed the lowest intake of fat and the most frequent intake of carotenoids among the three groups (data not shown). Typical for the men in the "High fat" group was a more frequent consumption of high-fat spreads, coffee, fat in cooking, high-fat milk, sugar and jam, and beer. Concurrently, these men exhibited the lowest intake of energy, protein, carbohydrates, alcohol, retinol, carotenoids, vitamin C, calcium, and iron among the groups (data now shown). Finally, the men in the "Tea, soda and cookies" group demonstrated more frequent consumption of soda, tea and cookies. These men had the highest intake of protein, fat, carbohydrates, vitamin C, calcium, and iron among the groups (data now shown).

Among women, typical characteristics of the "Fruit and vegetables" group were a higher proportion of Low Energy Reporters, as well as a higher mean age and a higher mean BMI and weight. Among the "High fat" group, typical characteristics included a higher proportion of smokers, lower education, and lower mean BMI and weight. Among the "Coffee and sandwich" group, common characteristics were a higher proportion of Low Energy Reporters, a higher proportion of smokers, and lower education. Finally, typical characteristics of the "Tea and ice cream" group were a lower mean age, a low proportion of smokers, and high education. These differences between groups were significant (*P *< 0.000).

Among men, typical characteristics of the "Fruit and vegetables" group included a higher proportion of Low Energy Reporters, and a higher mean BMI and weight. Among the "High fat" group, typical characteristics were a higher proportion of Low Energy Reporters and a higher proportion of smokers, as well as a higher proportion of snuff users, and lower education. Finally, among members of the "Tea, soda and cookies" group, typical features included lower age and being unmarried. These differences in mean characteristics between groups were significant (*P *< 0.000).

## Discussion

The strength of the VIP dietary database is its size, with over 60,000 records between 1992–2005. The most suitable dietary data collection tool for such large epidemiological studies is the FFQ, which provides approximations of habitual diet over longer periods of time, and may allow ranking of individuals according to food or nutrient intake [14]. The FFQ is simple and user-friendly but, unfortunately, has limitations. The large proportion of Low Energy Reporters among both women and men in our study illustrates one common limitation of FFQs. Even so, our levels are higher than those commonly reported. This may reflect the relatively limited number of food items in our FFQ (84 and 64), because reported intake levels tend to correlate with number of food items available in the FFQ [14]. In accordance with other studies, low energy reporting in our study was more common among those with higher BMI, higher age, and lower education [15-17]. Thus, results from the VIP dietary database should be interpreted with caution in comparisons with recommended levels of intake. However, our validation study demonstrated the database's ability to correctly rank individuals and it is for this purpose that we recommend that the VIP dietary database be used in the future. Consequently, our results on food intake patterns, which are based on rankings, should still be valid. This is supported by our comparison of results from cluster analyses on the entire sample (as shown in this paper) and cluster analyses only on plausible reporters (see Methods above). Similar conclusions also were reached in an American comparison of dietary patterns based on the entire study sample and based only on plausible reporters [18].

To improve robustness and account for differences in reported energy intake, our analyses were based on reported frequency of intake/1000 kcalories. Groups with high levels of underreporting, such as the "Fruit and vegetables" groups, may thus be represented by higher frequencies/1000 kcalories than other groups with lower levels of underreporting. This may falsely enhance differences among groups in the cluster analyses process. However, reported differences in frequency of intake among cluster groups were indeed large even without energy adjustment, as seen in Tables 3 and 4.

On the whole, women reported significantly more frequent consumption of fruit and vegetables compared with men, consistent with earlier findings from the Swedish national dietary study [19]. Indeed, in our study the female cluster with the least frequent intake reported 40% more frequent consumption of fruits and 26% more frequent consumption of vegetables than the male cluster with the most frequent intake. The Swedish national dietary study [19] found higher intakes of fruit and vegetables among women than among men. In our study, men had a significantly more frequent consumption of sugar and jam, beer, and spirits compared with women, also this consistent with the Swedish national dietary study [19]. Consequently, most patterns by sex found in our study are in line with national data.

Women and men exhibiting a "Fruit and vegetables" eating pattern in general reported a healthy diet, with frequent consumption of fruit and vegetables and low-fat products. A typical characteristic of "Fruit and vegetables" women and men was a higher proportion of underreporting, which may reflect a desire to report only what is healthy. Thus, it is possible that the profile of healthy eating in these groups partly reflects a desire to eat healthy and partly what is actually being consumed. Similarly, the higher BMI values in these groups may reflect this desire to eat healthy or it may reflect a pre-existing condition that has encouraged these women and men to change to a healthier diet.

Women and men with a "High fat" diet had frequent consumption of high-fat products, beer and sugar. "High fat" women and men typically were smokers and had a lower educational level. Other studies have also reported clusters with distinct healthy and non-healthy profiles, e.g., among Americans [20], Italians [21], and Irish individuals [22]. Of these studies, only the Italian one developed clusters for women and men separately. Hence, cluster membership can probably be said to capture a broad lifestyle profile. This will be further explored in a forthcoming paper.

Among women in our study, two additional distinct patterns were seen, the "Coffee and sandwich" and "Tea and ice cream" clusters, while among men, a third group emerged, labeled "Tea, soda and cookies". Women in the "Coffee and sandwich" group had frequent consumption of coffee, bread and low-fat products, while women in the "Tea and ice cream" group and men in the "Tea, soda and cookies" group reported frequent consumption of the food items indicated in their names. Such patterns have not been reported in studies from other countries using cluster analyses and this may reflect consumption patterns more typical for Sweden. A study of food clusters among adults (both sexes together) in southern Sweden identified one cluster with "Sweets and cakes" and one with "White bread" [23]; these may correspond to our "Coffee and sandwich" group.

In our analyses, excluded individuals reported higher body mass index and less healthy life style habits than did included individuals; a finding consistent with most studies evaluating this phenomenon. However, due to our large sample size, even small differences in background variables exhibited large statistical significance. Still, future analyses of associations between dietary patterns and health outcomes need to take possible consequences of selection bias into account. Finally, it was not within the scope of this analysis to evaluate changes in intake that may have occurred during the data collection period. However, this would be valuable to further investigate in the future.

## Conclusion

In conclusion, more distinct food patterns, including larger variation in frequency of daily intake, was found among women than among men for most food groups. Due to the high level of low energy reporting, our results on dietary intake may not be suitable for comparisons with recommended intake levels. However, the results on food intake patterns should be valid and useful as a basis for targeting interventions to groups most in need.

## Abbreviations

BMI: body mass index; BMR: basal metabolic rate; CI: confidence interval; FFQ: food frequency questionnaire; FIL: food intake level; PAL: physical activity level; and VIP: Västerbotten Intervention Program.

## Competing interests

The authors declare that they have no competing interests.

## Authors' contributions

GH, BL, LW and IJ designed and carried out the Västerbotten Intervention Project and the collection of dietary data. IJ was responsible for the validation of dietary data. AW and IJ were responsible for the creation of a coherent dietary data base. AW and AH carried out the statistical analysis. AW drafted the manuscript. All authors read and approved the final manuscript.

## Supplementary Material

Additional file 1 **Reported intake of important macro- and micronutrients among study women and men by age groups.** As supplementary information, we present four tables on intake of macro- and micronutrients among women and men in northern Sweden in 1992–2005, by age. Many nutrients exhibit a skewed distribution; hence, both means and standard deviations (SDs) are included. Click here for file
