# Supplementary material for: More distinct food intake patterns among women than men in northern Sweden: a population-based survey
Source: Nutr J. 2009 Feb 19;8:12. doi: 10.1186/1475-2891-8-12 (PMC2649165; doi:10.1186/1475-2891-8-12)
Supplement: Additional file 1 — Reported intake of important macro- and micronutrients among study women and men by age groups. As supplementary information, we present four tables on intake of macro- and micronutrients among women and men in northern Sweden in 1992–2005, by age. Many nutrients exhibit a skewed distribution; hence, both means and standard deviations (SDs) are included. [file 1475-2891-8-12-S1.doc]

**Supplementary Table 1. Intake of macronutrients among women in northern Sweden by age group**

| **Age (yrs)** | **n** | **Energy (MJ)** | **Protein (g)** | **Fat (g)** | **Carbohydrate (g)** | **Alcohol (g)** |
| --- | --- | --- | --- | --- | --- | --- |
| 30 | 3 049 | 8.05^1^ + 1.93  7.94 (6.64-9.35) | 67.0 + 17.4  65.5 (54.5 - 77.6) | 75.4 + 23.6  75.7 (60.8 - 93.4) | 231.1 + 62.5  226.1 (185.3-271.3) | 2.91 + 2.16  2.65 (1.54 - 4.06) |
| 40 | 10 516 | 6.97 + 1.69  6.79 (5.70-8.05) | 61.9 + 16.6  60.3 (49.9 - 72.1) | 61.0 + 17.6  58.8 (48.4 - 71.2) | 210.0 + 58.2  203.3 (167.4-245.8) | 3.50 + 2.74  3.12 (1.80 - 4.54) |
| 50 | 10 851 | 6.88 + 1.77  6.67 (5.56-8.00) | 57.6 + 15.6  55.6 (46.3 - 66.9) | 55.1 + 16.3  53.1 (43.4 - 64.5) | 197.6 + 55.3  191.2 (157.2-230.7) | 3.32 + 2.92  2.89 (1.47 - 4.26) |
| 60 | 8 184 | 6.13 + 1.49  5.89 (5.02-6.98) | 55.5 + 15.0  53.3 (44.9 - 63.7) | 49.4 + 15.0  47.0 (38.8 - 57.3) | 193.8 + 52.9  186.4 (155.1-224.6) | 3.09 + 3.19  1.96 (0.67 -3.86) |
| All | 32 600 | 6.83 + 1.77  6.60 (5.49-7.94) | 59.3 + 16.3  57.1 (47.4 - 69.0) | 57.7 + 19.0  54.7 (44.1 - 68.2) | 203.8 + 57.4  196.4 (161.2-238.4) | 3.19 + 2.86  2.76 (1.30 - 4.32) |

^1^ x + sd; median (25^th^ – 75^th^ percentile)

**Supplementary Table 2. Intake of macronutrients among men in northern Sweden by age group**

| **Age**  **(yrs)** | **n** | **Energy (MJ)** | **Protein (g)** | **Fat (g)** | **Carbohydrate (g)** | **Alcohol (g)** |
| --- | --- | --- | --- | --- | --- | --- |
| 30 | 2 667 | 10.12^1^ + 2.34  10.09 (8.41-11.83) | 85.7 + 22.3  83.9 (69.7 - 99.9) | 101.6 + 29.8  99.5 (80.2-120.6) | 276.6 + 72.5  273.8 (223.7-326.3) | 7.44 + 4.99  6.82 (4.14 - 9.71) |
| 40 | 9 580 | 8.66 + 2.23  8.38 (6.96-10.08) | 73.4 + 20.4  70.7 (58.2 - 85.3) | 83.3 + 25.7  79.4 (64.4 - 98.5) | 237.0 + 71.4  227.9 (184.6-280.2) | 6.07 + 4.77  5.19 (3.08 - 7.71) |
| 50 | 10 157 | 8.53 + 2.19  8.27 (6.86-9.95) | 71.7 + 20.2  69.2 (56.9 - 84.0) | 80.2 + 25.3  76.5 (62.0 - 94.4) | 243.7 + 73.1  235.4 (188.8-289.6) | 7.03 + 5.49  5.99 (3.44 - 9.03) |
| 60 | 7 527 | 8.53 + 2.18  8.27 (6.86-9.97) | 72.7 + 20.4  70.4 (58.1 - 85.3) | 77.0 + 24.4  73.5 (59.2 - 90.5) | 250.6 + 72.6  242.3 (196.6-297.2) | 7.22 + 6.98  5.84 (2.80 - 9.76) |
| All | 29 931 | 8.71 + 2.26  8.44 (6.99-10.19) | 73.7 + 20.8  71.2 (58.4 - 86.3) | 82.3 + 26.4  78.3 (62.8 - 97.5) | 246.2 + 73.2  237.9 (191.5-292.3) | 6.81 + 5.68  5.75 (3.27 - 8.87) |

^1^ x + sd; median (25^th^ – 75^th^ percentile)

**Supplementary Table 3. Intake of micronutrients among women in northern Sweden by age group**

| **Age (yrs)** | **n** | **Iron (mg)** | **Calcium (mg)** | **Vitamin C (mg)** | **Retinol (mg)** | **Carotenoid (mg)** |
| --- | --- | --- | --- | --- | --- | --- |
| 30 | 3 049 | 14.9 + 4.3  14.4 (11.8 - 17.5) | 889 + 328  859 (653-1083) | 91.7 + 53.6  80.1 (53.3-116.0) | 0.92 + 0.45  0.84 (0.63- 1.10) | 4.15 + 4.15  2.81 (1.52- 5.46) |
| 40 | 10 516 | 12.5 + 3.7  12.1 (9.8 - 14.7) | 781 + 315  742 (549- 975) | 75.0 + 45.6  63.8 (44.1- 94.6) | 0.60 + 0.28  0.54 (0.41- 0.72) | 5.34 + 5.32  3.82 (1.87- 7.63) |
| 50 | 10 851 | 11.3 + 3.4  10.9 (8.8 - 13.3) | 746 + 290  712 (537- 918) | 75.6 + 42.9  65.4 (46.2- 94.5) | 0.57 + 0.27  0.51 (0.39- 0.68) | 5.50 + 5.42  3.91 (1.97- 7.87) |
| 60 | 8 184 | 10.8 + 3.3  10.3 (8.3 - 12.7) | 733 + 273  701 (539-894) | 73.3 + 42.5  62.9 (44.4- 91.2) | 0.51 + 0.28  0.45 (0.34- 0.62) | 5.43 + 5.17  3.95 (2.14- 7.79) |
| All | 32 600 | 11.9 + 3.8  11.3 (9.1 - 14.1) | 768 + 301  729 (548- 946) | 76.3 + 45.0  65.3 (45.5- 95.6) | 0.60 + 0.32  0.53 (0.39- 0.72) | 5.31 + 5.23  3.80 (1.90- 7.66) |

^1^ x + sd; median (25^th^ – 75^th^ percentile)

**Supplementary Table 4. Intake of micronutrients among men in northern Sweden by age group**

| **Age**  **(yrs)** | **n** | **Iron (mg)** | **Calcium (mg)** | **Vitamin C (mg)** | **Retinol (mg)** | **Carotenoid (mg)** |
| --- | --- | --- | --- | --- | --- | --- |
| 30 | 2 667 | 17.7^1^ + 4.9  17.4 (14.1-20.7) | 1135 + 451  1096 (786-1434) | 72.5 + 41.0  63.5 (43.5- 92.2) | 1.16 + 0.57  1.07 (0.80- 1.41) | 2.47 + 2.35  1.69 (1.05- 2.87) |
| 40 | 9 580 | 14.6 + 4.3  14.0 (11.4-17.2) | 872 + 352  834 (607-1088) | 80.7 + 49.1  69.0 (47.0-100.6) | 0.76 + 0.33  0.71 (0.54- 0.92) | 2.88 + 2.95  1.87 (1.15- 3.59) |
| 50 | 10 157 | 14.5 + 4.4  13.9 (11.2-17.1) | 877 + 358  839 (605-1088) | 85.2 + 50.3  74.8 (49.9-106.5) | 0.80 + 0.39  0.72 (0.54-0.97) | 3.03 + 3.16  1.98 (1.19- 3.69) |
| 60 | 7 527 | 14.7 + 4.6  14.0 (11.3-17.5) | 932 + 363  895 (665-1148) | 86.9 + 50.3  77.4 (50.9-108.2) | 0.80 + 0.46  0.69 (0.52- 0.94) | 3.17 + 3.43  2.14 (1.17- 3.93) |
| All | 29 931 | 14.9 + 5.0  14.2 (11.5-17.7) | 912 + 374  868 (633-1135) | 83.1 + 49.3  72.4 (48.4-104.0) | 0.82 + 0.42  0.73 (0.55- 0.99) | 2.97 + 3.11  1.92 (1.16- 3.65) |

^1^ x + sd; median (25^th^ – 75^th^ percentile)
